# Effect of central sensitization on postoperative cognitive dysfunction in total knee arthroplasty patients: a retrospective study

**DOI:** 10.3389/fneur.2024.1509197

**Published:** 2025-01-15

**Authors:** Qiqi Yang, Chunning Li, Min Ye, Xinhua Zhou, Weiran Li, Fei Li

**Affiliations:** ^1^Second Affiliated Hospital of Anhui University of Chinese Medicine, Hefei, China; ^2^The First Clinical Medical School, Anhui University of Chinese Medicine, Hefei, China; ^3^First Affiliated Hospital of Bengbu Medical College, Bengbu, China; ^4^Hefei Southeast Orthopedics Hospital, Hefei, China

**Keywords:** central sensitization, postoperative cognitive dysfunction, total knee arthroplasty, retrospective study, Mini-Mental State Examination, Knee Injury and Osteoarthritis Outcome Score

## Abstract

**Background:**

Postoperative cognitive dysfunction (POCD) is a common complication after total knee arthroplasty (TKA), impacting recovery and quality of life. This study aims to investigate central sensitization (CS) as an independent risk factor for POCD to improve preoperative screening and postoperative interventions.

**Methods:**

A retrospective analysis was conducted on 142 TKA patients from January 2020 to May 2024 across three hospitals. Data were collected at six time points: preoperatively (T0), intraoperatively (T1), and postoperatively on days 1 (T2), 3 (T3), 7 (T4), and 30 (T5). Patients were classified into CS (CSI ≥ 40) and non-CS (CSI < 40) groups according to Central Sensitization Inventory (CSI) score. Cognitive function and POCD incidence were assessed with the Mini-Mental State Examination (MMSE), and knee recovery with the Knee Injury and Osteoarthritis Outcome Score (KOOS). Logistic regression was used to identified risk factors for POCD.

**Results:**

The overall incidence of POCD at T5 was 19.72%, with a significantly higher rate in CS group (30.91%) compared to non-CS group (12.64%) (*p* = 0.008). MMSE scores declined significantly in both groups at T2 and T3 compared to T0 (*p* < 0.05), with CS group showing consistently lower scores than non-CS group at T2-T5 (*p* < 0.05). KOOS scores revealed that CS group had worse pain and quality of life scores at T0, T4, and T5 compared with non-CS group (*p* < 0.05). Logistic regression revealed that CS, cerebrovascular disease, intraoperative hemorrhage, and preoperative MMSE were associated with the risk of POCD (*p* < 0.05).

**Conclusion:**

CS is a significant risk factor for POCD following TKA, adversely affecting recovery in terms of pain and quality of life. Prospective studies are warranted to validate findings and develop targeted interventions.

## 1 Introduction

Total Knee Arthroplasty (TKA) is widely regarded as the most effective surgical intervention for managing end-stage knee osteoarthritis (KOA), offering significant pain relief and functional restoration (1, 2). Despite advancements in surgical techniques, prosthetic knee designs (3), and postoperative rehabilitation protocols, approximately 10–40% of patients may still develop postoperative cognitive dysfunction (POCD) (4, 5). POCD is a common postoperative syndrome characterized by cognitive impairments, including deficits in memory, intellectual ability, and executive function (6). This complex condition often persists beyond the expected recovery period from anesthesia, contributing to prolonged hospital stays, increased healthcare costs, greater care requirements, as well as elevated morbidity and mortality (7–9).

The precise mechanisms underlying POCD remain poorly understood, emerging evidence points to a potential role of central sensitization (CS) in its pathogenesis (10). CS, a pathological condition frequently observed in chronic pain patients, is marked by a heightened excitability of the central nervous system (CNS) triggered by persistent peripheral nociceptive stimuli, leading to exaggerated pain responses even from subthreshold or normal sensory input (11). CS has been shown to a significant risk factor for persistent pain and patient dissatisfaction after TKA (12, 13). However, CS not only intensifies pain perception in patients undergoing TKA, but also potentially compromises cognitive function through a cascade of neuroinflammatory processes (14, 15). Studies suggest that patients with CS exhibit a significantly lowered pain threshold, a phenomenon closely linked to microglial activation and the release of pro-inflammatory cytokines such as IL-6 and TNF-*α* within the CNS (16–18). These inflammatory mediators are thought to be key drivers of cognitive decline (19, 20). This suggests that CS is not only a primary mechanism responsible for chronic postoperative pain but also a contributor to POCD through sustained neuroinflammation and altered neuroplasticity.

Notably, the presence of CS in TKA patients is often accompanied by preoperative chronic pain and psychosocial factors, such as anxiety, depression, and sleep disturbances, all of which are themselves closely linked to the occurrence of POCD (21, 22). As such, CS is considered to be a critical risk factor for POCD following TKA. Although previous studies have explored the impact of CS on postoperative pain and functional recovery, systematic research into its relationship with POCD remains very limited.

Given this, the study aims to conduct a retrospective analysis to investigate the impact of preoperative CS on POCD in TKA patients. Patients will be stratified based on their preoperative Central Sensitization Inventory (CSI) scores, and the correlation between CS and POCD will be assessed. The findings from this study are expected to offer new insights for preoperative screening, and postoperative management, ultimately reducing the incidence of POCD and improving patient outcomes.

## 2 Materials and methods

### 2.1 Study design

This study is a multi-center, retrospective cohort investigation aimed at evaluating the impact of CS on POCD in patients undergoing TKA. Clinical data were sourced from medical records at the First Affiliated Hospital of Bengbu Medical College, the First Affiliated Hospital of Anhui University of Traditional Chinese Medicine, and Hefei Southeast Orthopedics Hospital. The study adhered to the STROBE guidelines and received ethical approval from the Institutional Review Board (2023-zj-40). As all data were de-identified, informed consent from patients was waived.

### 2.2 Participants

This study retrospectively analyzed patients who underwent TKA at the aforementioned three hospitals between January 1, 2020, and May 31, 2024. The inclusion criteria were as follows: (1) age ≥ 55 years; (2) diagnosed with end-stage KOA; (3) undergoing primary unilateral TKA; and (4) having complete CSI scores. Exclusion criteria included: (1) simultaneous bilateral TKA or revision TKA; (2) a history of central nervous system or psychiatric disorders; (3) severe sensory impairments affecting vision or hearing; (4) severe comorbidities involving the heart, brain, or lungs; (5) preoperative Mini-Mental State Examination (MMSE) score ≤ 23; (6) use of cognitive-impairing medications pre- or post-surgery (e.g., sedatives, anti-inflammatory drugs, psychotropics); and (7) incomplete clinical records.

### 2.3 Data collection

We collected comprehensive demographic and clinical data from patients meeting the study criteria, including age, gender, BMI, educational level, American Society of Anesthesiologists (ASA) classification, smoking, drinking, duration of KOA, and comorbidities such as hypertension, hyperlipidemia, diabetes, coronary heart disease, cerebrovascular disease, and chronic obstructive pulmonary disease. Data were collected at the following time points: preoperatively (T0), intraoperatively (T1), on postoperative day 1 (T2), postoperative day 3 (T3), postoperative day 7 (T4), and postoperative day 30 (T5). At T0, CSI scores were recorded to assess the state of CS, and preoperative hemoglobin levels were documented to evaluate the presence of anemia. At T1, the anesthesia methods, duration of anesthesia, duration of surgery, and intraoperative blood loss were documented. At T2-T5, MMSE scores were recorded to assess cognitive function and determine the incidence of POCD, while the Knee Injury and Osteoarthritis Outcome Score (KOOS) was used to evaluate postoperative recovery. Although MMSE is not universally a routine clinical assessment item for TKA patients, its inclusion in this study reflects institutional efforts to systematically monitor and manage POCD. The MMSE data were collected as part of the standard cognitive assessments implemented at the participating institutions and were consistently recorded in the hospitals’ medical records by trained clinical staff.

### 2.4 Assessment tools

#### 2.4.1 Central sensitization assessment

The Central Sensitization Inventory (CSI) was used preoperatively to assess the CS status of patients. The CSI is a validated, patient-reported questionnaire designed to evaluate symptoms associated with CS. It has been demonstrated to be a reliable, consistent, and effective tool for quantifying the severity of CS-related symptoms (23). The inventory includes 25 items, each scored from 0 to 4, with a total score ranging from 0 to 100. Based on established criteria (24, 25), a score of 40 or higher indicates the presence of CS, while a score below 40 suggests the absence of CS. In this study, patients were categorized into a CS group (CSI ≥ 40) and a non-CS group (CSI < 40).

#### 2.4.2 Cognitive function assessment

The MMSE scores was used to evaluated cognitive function (26). It primarily evaluates five cognitive domains: orientation, registration, attention and calculation, recall, and language abilities. The MMSE consists of 24 items with a total score of 30, where higher scores indicate better cognitive function. In this study, POCD was defined based on the changes in MMSE scores observed on postoperative day 30 (T5). Specifically, POCD was defined as a decline of 1 or more standard deviations (SD) in the postoperative MMSE score compared to the preoperative score. A reduction of 1–2 SD indicates postoperative mild neurocognitive disorder (NCD), while a reduction greater than 2 SD indicates postoperative major neurocognitive disorder (27).

#### 2.4.3 Knee function assessment

The KOOS is a patient-reported outcome measurement system used to evaluate short-term and long-term symptoms and function in individuals after TKA. The score consisted of five separately scored subscales: pain, symptoms, function in daily living (ADL), function in sport and recreation (Sport/Rec), and knee-related quality of life (QoL) (28). The KOOS scale consists of 42 items with a total score ranging from 0 to 100, where higher scores indicate better functional recovery.

### 2.5 Statistical analysis

All statistical analyses were performed using SPSS version 26.0. Continuous variables are presented as mean ± standard deviation (SD), while categorical variables are expressed as frequencies and percentages. For continuous variables measured at multiple time points (e.g., MMSE and KOOS scores at preoperative and various postoperative intervals), repeated measures analysis of variance (ANOVA) was employed to assess both within-group and between-group trends. Independent samples t-tests were conducted to compare mean values between two groups, whereas paired t-tests were utilized to evaluate changes within the same group at two distinct time points. Categorical variables were analyzed using the chi-square test. To identify potential risk factors for POCD, both univariate and multivariate logistic regression analyses were performed, and odds ratios (ORs) with corresponding 95% confidence intervals (CIs) were calculated. All statistical tests were conducted with a significance level set at *p* < 0.05.

## 3 Results

### 3.1 Patient demographics and clinical characteristics

**Figure 1:**
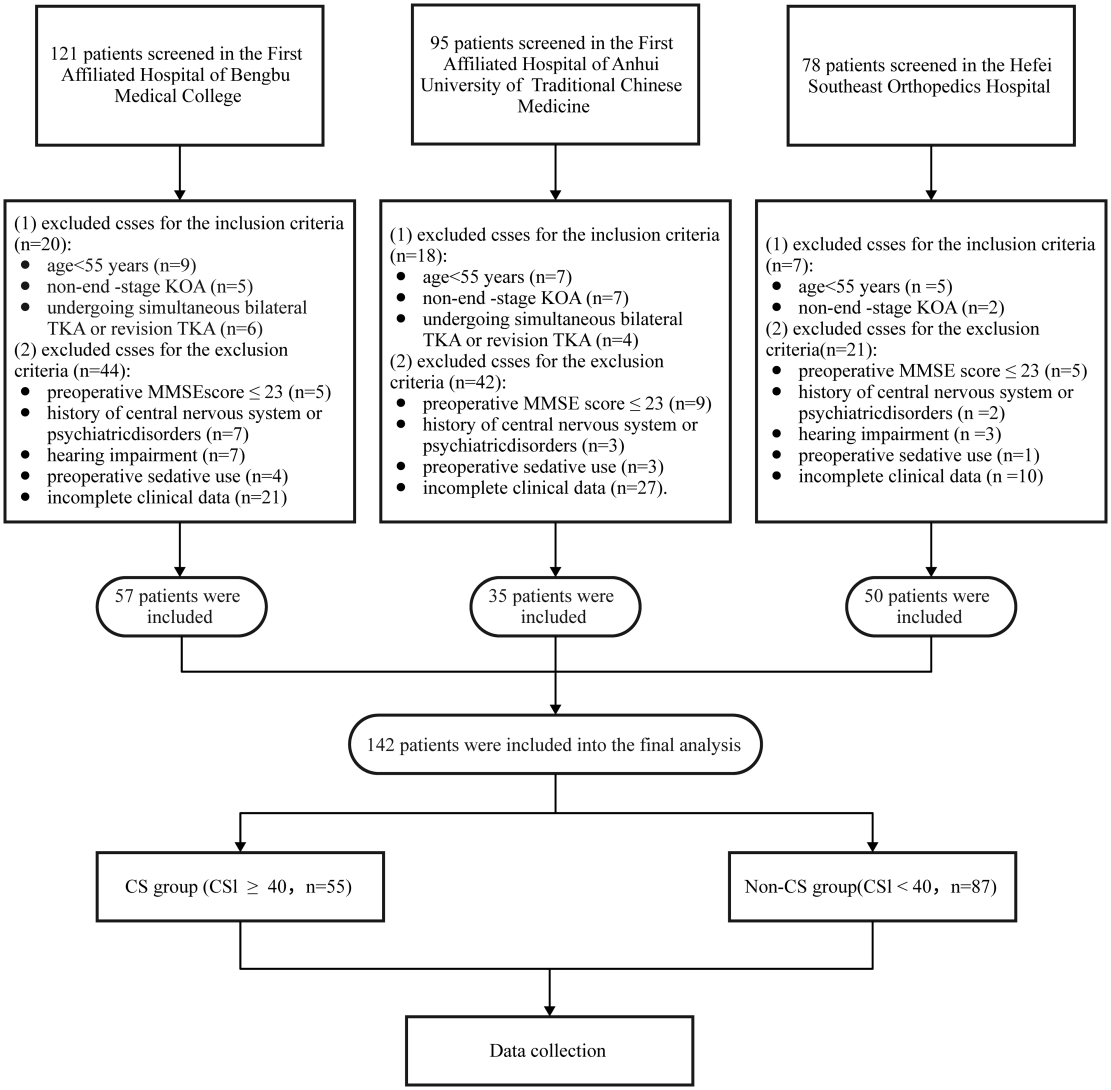
Flow chart of patient selection. TKA, total knee arthroplasty; KOA, Knee osteoarthritis; CS, central sensitization; MMSE, mini-mental state examination.

A total of 294 patients scheduled for TKA were initially recruited for the study. However, 152 patients were excluded based on the following criteria: age < 55 years, revision TKA, non-end-stage KOA, preoperative MMSE score < 24, history of psychiatric disorders, hearing impairment, preoperative sedative use, and incomplete clinical data (*n* = 54). Ultimately, 142 patients were included in the final analysis. Figure 1 illustrates the patient selection flowchart.

Among the 142 patients enrolled in the study, 55 were assigned to the CS group (CSI ≥ 40), and 87 to the non-CS group (CSI < 40). Analysis of baseline data revealed no significant differences between the groups in terms of age, gender, BMI, education level, smoking, drinking, ASA classification, duration of KOA, comorbidities, preoperative hemoglobin, anemia, preoperative MMSE, operation time, intraoperative hemorrhage, anesthesia type, and anesthesia time (all *p* > 0.05) (Table 1).

**Table 1:** Comparison of baseline demographic and clinical characteristics.

Variables	CS group (*n* = 55)	Non-CS group (*n* = 87)	*t/χ^2^*	*p* value
Age, years (^−^x ± s)	67.07 ± 5.89	65.64 ± 4.82	1.579	0.117
Gender, *n* (%)				
Male	26 (47.27%)	45 (51.72%)	0.267	0.605
Female	29 (52.73%)	42 (48.28%)	0.267	0.605
BMI, kg/m^2^ (^−^x ± s)	26.31 ± 3.98	27.24 ± 3.68	1.416	0.159
Educational level, *n* (%)				
Below high school	37 (67.27%)	47 (54.02%)	2.448	0.118
High school and above	18 (32.73%)	40 (45.98%)	2.448	0.118
Smoking, *n* (%)	13 (23.64%)	27 (31.03%)	0.912	0.340
Drinking, *n* (%)	19 (34.55%)	30 (34.48%)	0.912	0.994
ASA classification, *n* (%)				
I	7 (12.73%)	11 (12.64%)	0.0002	0.989
II	38 (69.09%)	53 (60.92%)	0.978	0.323
III	10 (18.18%)	23 (26.44%)	1.287	0.257
Duration of KOA, years (^−^x ± s)	9.71 ± 7.81	8.20 ± 6.59	1.237	0.218
Combined underlying diseases, *n* (%)				
Hypertension	22 (40.00%)	27 (31.03%)	1.199	0.274
Hyperlipidemia	10 (18.18%)	16 (18.39%)	0.004	0.950
Diabetes	14 (25.45%)	25 (28.74%)	0.450	0.502
Coronary heart disease	7 (12.73%)	6 (6.90%)	1.378	0.241
Cerebrovascular disease	5 (9.09%)	11 (12.64%)	0.425	0.514
Chronic obstructive pulmonary	11 (20.00%)	19 (21.84%)	0.068	0.794
Preoperative hemoglobin, g/L (^−^x ± s)	132.55 ± 14.99	132.93 ± 18.56	0.128	0.899
Anemia, *n* (%)	3 (5.45%)	3 (3.45%)	0.023	0.880
Preoperative MMSE, scores (^−^x ± s)	27.84 ± 1.10	28.10 ± 0.85	1.624	0.107
Operation time, *n* (%)				
<120 min	12 (21.82%)	26 (29.89%)	1.119	0.290
≥120 min	43 (78.18%)	61 (70.11%)	1.119	0.290
Intraoperative hemorrhage, *n* (%)				
<200 mL	11 (20.00%)	21 (24.14%)	0.331	0.565
≥200 mL	44 (80.00%)	66 (75.86%)	0.331	0.565
Anesthesia type, *n* (%)				
General anesthesia	31 (56.36%)	45 (51.72%)	0.403	0.526
Neuraxial anesthesia	19 (34.55%)	28 (32.18%)	0.085	0.771
Combination anesthesia	5 (9.09%)	14 (16.09%)	1.425	0.233
Anesthesia time, *n* (%)				
<150 min	13 (23.64%)	24 (27.59%)	0.273	0.601
≥150 min	42 (76.36%)	63 (72.41%)	0.273	0.601

CS, central sensitization; BMI, body mass index; ASA, American society of anesthesiologists; KOA, knee osteoarthritis; MMSE, Mini-Mental State Examination.

### 3.2 Comparison of the incidence of POCD

**Figure 2:**
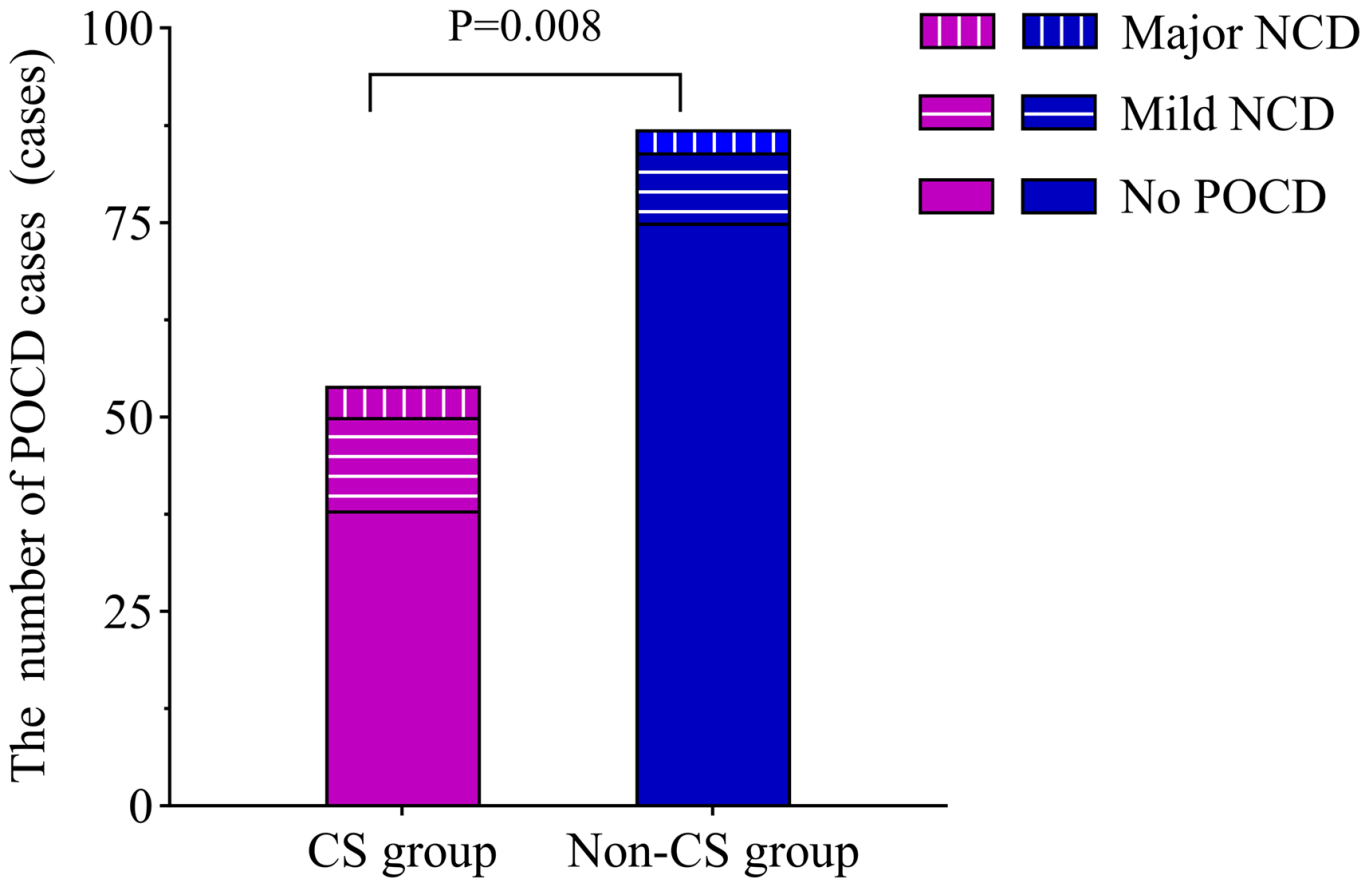
Comparison of POCD cases on postoperative day 30. The incidence of POCD was compared between the CS and non-CS groups using the chi-square test. CS, central sensitization; POCD, postoperative cognitive dysfunction; NCD, neurocognitive disorder.

The overall incidence of POCD on 30 days postoperatively was 19.72% (28/142). The incidence in the CS group was 30.91% (17/55), significantly higher than 12.64% (11/87) in the non-CS group (*p* = 0.008), indicating a strong correlation between central sensitization and the overall occurrence of POCD. However, when examining the severity of POCD, no significant differences were observed between the two groups regarding the proportions of mild and major cases (*p*>0.05). In the CS group, 12 cases were classified as postoperative mild NCD and 5 as major, while in the non-CS group, 9 cases were mild and 2 were postoperative major NCD. These findings suggest that central sensitization primarily influences the overall incidence of POCD rather than its severity (Figure 2).

### 3.3 Comparison of MMSE scores

Repeated measures ANOVA indicated a significant main effect of group on MMSE scores (*F* = 45.08, *p* < 0.001), indicating a notable difference in cognitive recovery between the CS group and the non-CS group. The main effect of time was also significant (*F* = 60.68, *p* < 0.001), reflecting a trend of change in MMSE scores over time. Moreover, the interaction between time and group was significant (*F* = 3.443, *p* = 0.009), suggesting that the two groups exhibited different trends in postoperative cognitive recovery.

**Figure 3:**
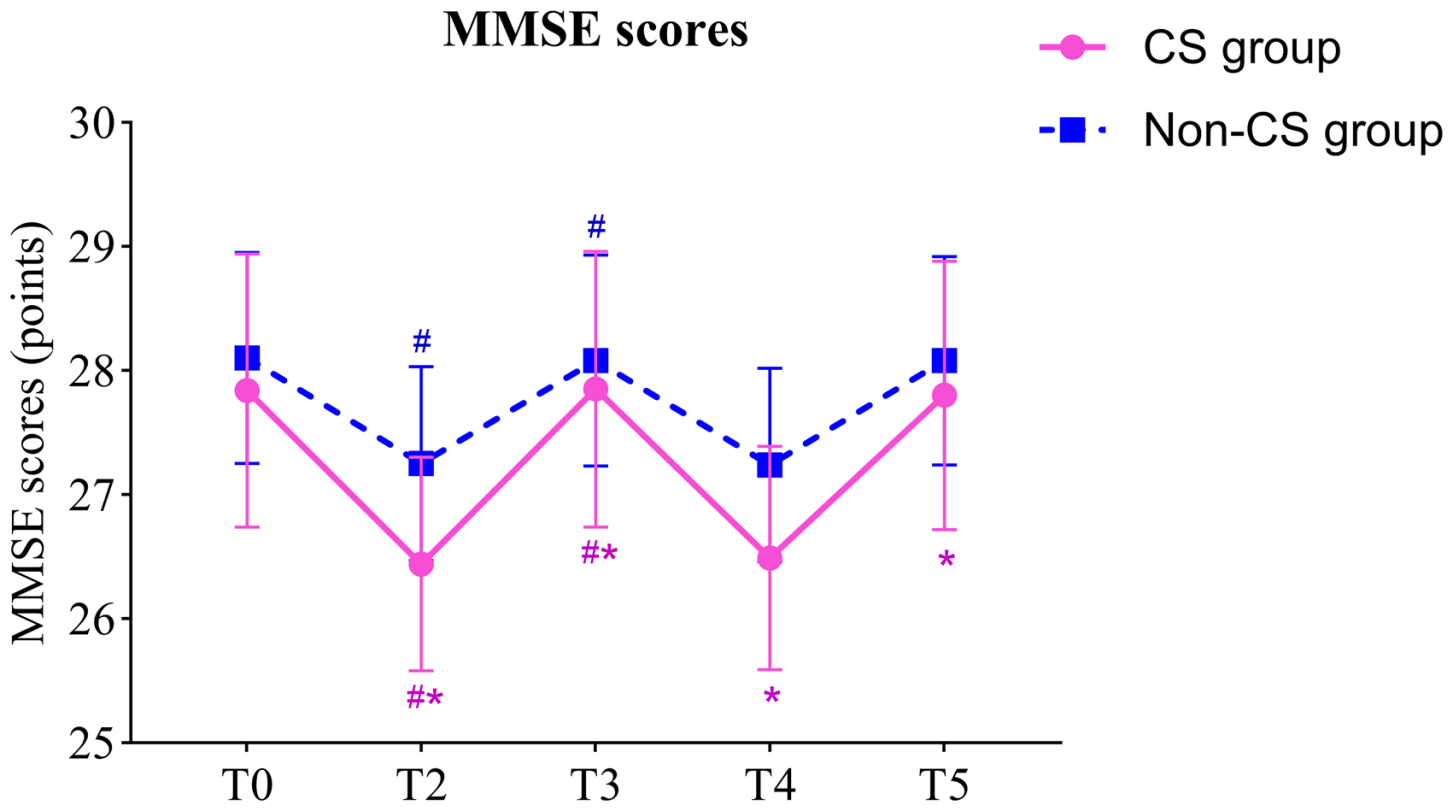
Comparison of MMSE scores between two groups. ^#^ indicates that compared with T0 (Paired t-tests), *p* < 0.05; ^*^ indicates comparison with non-CS group (Independent samples t-tests), *p* < 0.05; T0, preoperatively; T2, postoperative day 1; T3, postoperative day 3; T4, postoperative day 7; T5, postoperative day 30. MMSE, mini-mental state examination.

Paired comparisons within each group showed significant differences in MMSE scores for both the CS group and the non-CS group at T2 and T3 compared to T0 (*p* < 0.05). Between-group comparisons revealed significant differences in MMSE scores at all time points except T0 (*p* < 0.05). These findings suggest that patients in the CS group exhibited a marked delay in postoperative neurocognitive recovery, particularly during the early recovery phase (Table 2; Figure 3).

**Table 2:** Comparison of MMSE scores between two groups.

	T0	T2	T3	T4	T5
MMSE score					
CS	27.84 ± 1.10	26.44 ± 0.86	27.85 ± 1.11	26.49 ± 0.90	27.24 ± 0.78
NCS	28.10 ± 0.85	27.25 ± 0.78	28.08 ± 0.85	27.24 ± 0.78	28.08 ± 0.84
*t^a^*, *p* value*^a^*	1.624, 0.107	5.848, **<0.001**	5.131, **<0.001**	4.029, **<0.001**	4.365, **<0.001**
*t*^b^, *p* value^b^		7.903, **<0.001**	7.275, **<0.001**	1.937, 0.058	1.552, 0.126
*t*^**c**^, *p* value^**c**^		8.608, **<0.001**	9.061, **<0.001**	0.591, 0.556	0.910, 0.365

Bold indicates statistical significance *p* < 0.05.

T0, preoperatively; T2, postoperative day 1; T3, postoperative day 3; T4, postoperative day 7; T5, postoperative day 30; MMSE, Mini-Mental State Examination; CS, central sensitization.

^a^Statistical comparison between groups at each time point (Independent samples t-tests).

^b^Statistical comparison of different time points with the T0 time point in the CS group (Paired t-tests).

^c^Statistical comparison of different time points with the T0 time point in the Non-CS group (Paired t-tests).

### 3.4 Comparison of KOOS scores

Repeated measures ANOVA indicated a significant main effect of group on the pain and QoL subdomains of the KOOS scores (*F* = 85.26, *p* < 0.001; *F* = 85.88, *p* < 0.001), while no significant main effects were observed for symptoms, ADL, and Sport/Rec subdomains (*F* = 3.797, *p* = 0.052; *F* = 3.294, *p* = 0.07; *F* = 0.841, *p* = 0.360). This suggests that there were significant differences between the CS group and non-CS group in pain perception and quality of life recovery, but no significant differences in symptoms, daily activities, or sports/recreation. The main effect of time was significant across all five KOOS subdomains (*F* = 209.4, *p* < 0.001; *F* = 480.0, *p* < 0.001; *F* = 659.3, *p* < 0.001; *F* = 911.5, *p* < 0.001; *F* = 651.4, *p* < 0.001), indicating that patients experienced significant improvements in all KOOS subdomains over time. Furthermore, the interactions between time and group were observed for both pain and QoL scores (*F* = 10.74, *p* < 0.001; *F* = 6.201, *p* < 0.001), suggesting that the two groups exhibited different trends in postoperative pain relief and quality of life recovery.

**Figure 4:**
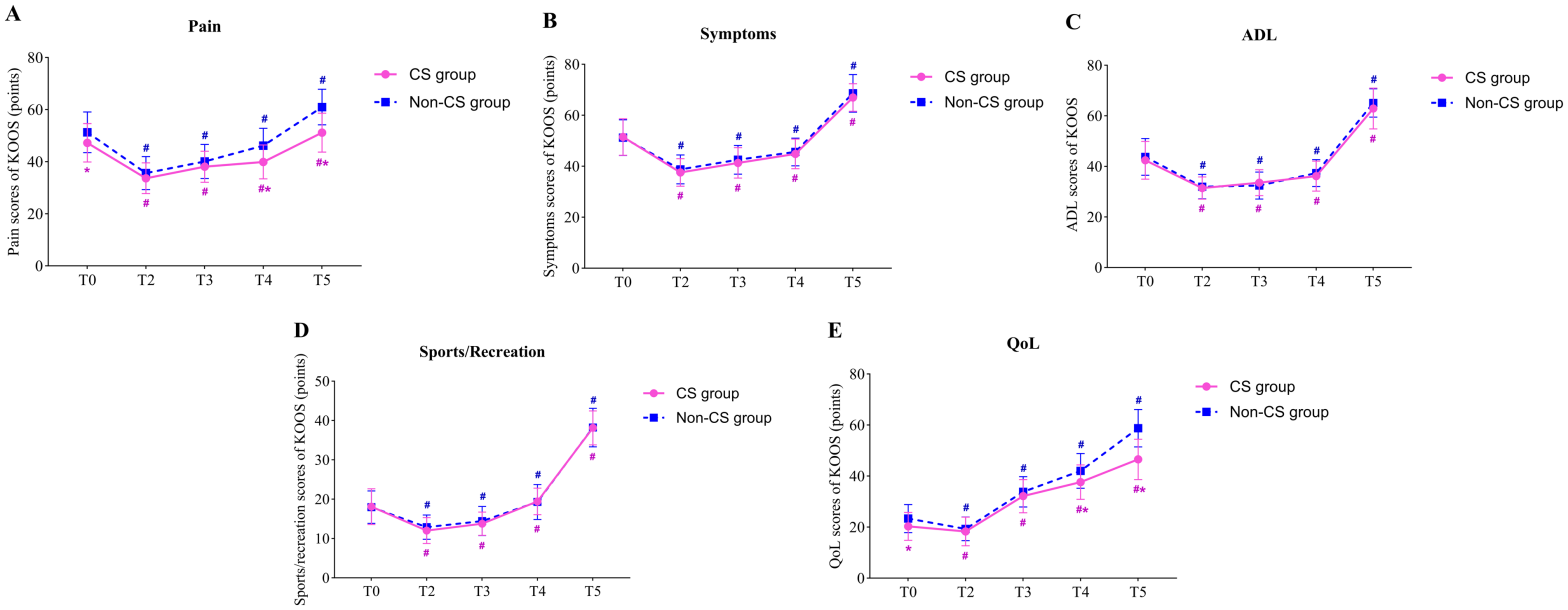
Comparison of KOOS scores between two groups. **(A)** the pain scores of KOOS; **(B)** the symptoms scores of KOOS; **(C)** the ADL scores of KOOS; **(D)** the sports/recreation scores of KOOS; **(E)** the QoL scores of KOOS; ^#^ indicates that compared with T0 (Paired t-tests), *p* < 0.05; ^*^ indicates comparison with non-CS group (Independent samples t-tests), *p* < 0.05; T0, preoperatively; T2, postoperative day 1; T3, postoperative day 3; T4, postoperative day 7; T5, postoperative day 30. KOOS, The Knee Injury and Osteoarthritis Outcome Score; ADL, activity of daily living; QoL, quality of life.

Paired comparisons within each group showed significant differences in all KOOS subdomains at all postoperative time points compared to T0 in both the CS and non-CS groups (*p* < 0.05), indicating overall recovery in pain, symptoms, daily activities, sports/recreation, and quality of life following TKA. Between-group comparisons revealed that the CS group had significantly lower pain and quality of life scores at T0, T4, and T5 compared to the non-CS group (*p* < 0.05), indicating a marked delay in postoperative pain relief and quality of life recovery in the CS group (Table 3; Figure 4).

**Table 3:** Comparison of KOOS scores between two groups.

	T0	T2	T3	T4	T5
KOOS Pain					
CS	47.24 ± 7.36	33.65 ± 5.89	38.07 ± 5.94	39.93 ± 6.50	51.18 ± 7.47
NCS	51.30 ± 7.82	35.62 ± 6.33	40.10 ± 6.53	46.18 ± 6.63	60.98 ± 6.83
*t^a^*, *p* value*^a^*	3.085, **0.002**	1.852, 0.067	1.868, 0.064	5.522, **<0.001**	8.029, **<0.001**
*t*^b^, *p* value^b^		12.51, **<0.001**	7.811, **<0.001**	5.702, **<0.001**	2.691, **0.010**
*t*^**c**^, *p* value^**c**^		17.80, **<0.001**	10.62, **<0.001**	4.478, **<0.001**	9.420, **<0.001**
KOOS Symptoms					
CS	51.47 ± 7.16	37.56 ± 5.40	41.33 ± 5.99	44.82 ± 5.78	66.96 ± 5.39
NCS	51.23 ± 6.97	38.78 ± 5.66	42.54 ± 5.64	45.61 ± 5.45	68.60 ± 7.35
*t^a^*, *p* value*^a^*	0.200, 0.842	1.272, 0.206	1.218, 0.225	0.823, 0.412	1.423, 0.157
*t*^b^, *p* value^b^		13.07, **<0.001**	7.842, **<0.001**	5.444, **<0.001**	12.19, **<0.001**
*t*^**c**^, *p* value^**c**^		17.83, **<0.001**	8.934, **<0.001**	5.793, **<0.001**	15.34, **<0.001**
KOOS ADL					
CS	42.43 ± 7.46	31.42 ± 4.39	33.55 ± 5.14	36.18 ± 5.92	62.91 ± 8.08
NCS	43.78 ± 7.27	31.97 ± 4.83	32.46 ± 5.33	37.36 ± 5.33	65.10 ± 5.60
*t^a^*, *p* value*^a^*	1.067,0.288	0.681, 0.497	1.205, 0.230	1.231, 0.220	1.907, 0.059
*t*^b^, *p* value^b^		10.24, **<0.001**	7.808, **<0.001**	5.223, **<0.001**	13.74, **<0.001**
*t*^**c**^, *p* value^**c**^		14.38, **<0.001**	11.58, **<0.001**	6.756, **<0.001**	22.29, **<0.001**
KOOS Sport/Rec					
CS	18.11 ± 4.54	12.01 ± 3.29	13.77 ± 2.97	19.43 ± 3.38	38.13 ± 4.31
NCS	17.98 ± 4.13	12.91 ± 3.08	14.45 ± 3.70	19.30 ± 4.43	38.21 ± 4.89
*t^a^*, *p* value*^a^*	0.183, 0.855	1.646,0.102	1.146, 0.254	0.180, 0.858	0.103, 0.918
*t*^b^, *p* value^b^		8.021, **<0.001**	5.744, **<0.001**	2.117, **0.039**	22.79, **<0.001**
*t*^**c**^, *p* value^**c**^		10.46, **<0.001**	6.557, **<0.001**	2.180, **0.032**	23.56, **<0.001**
KOOS QoL					
CS	19.63 ± 5.95	19.05 ± 6.28	32.36 ± 6.12	37.89 ± 6.42	47.27 ± 6.18
NCS	23.35 ± 5.50	20.72 ± 5.26	33.81 ± 5.94	42.04 ± 6.80	47.27 ± 6.18
*t^a^*, *p* value*^a^*	2.713, **0.008**	1.188, 0.237	1.404, 0.163	3.619, **<0.001**	9.644, **<0.001**
*t*^b^, *p* value^b^		2.571, **0.013**	11.83, **<0.001**	16.67, **<0.001**	21.60, **<0.001**
*t*^**c**^, *p* value^**c**^		6.044, **<0.001**	11.82, **<0.001**	20.91, **<0.001**	35.42, **<0.001**

Bold indicates statistical significance *p* < 0.05.

T0, preoperatively; T2, postoperative day 1; T3, postoperative day 3; T4, postoperative day 7; T5, postoperative day 30; CS, central sensitization; KOOS, Knee Injury and Osteoarthritis Outcome Score; Rec, Recreation; ADL, activity of daily living; QoL, quality of life.

^a^Statistical comparison between groups at each time point (Independent samples t-tests).

^b^Statistical comparison of different time points with the T0 time point in the CS group (Paired t-tests).

^c^Statistical comparison of different time points with the T0 time point in the Non-CS group (Paired t-tests).

### 3.5 Univariate and multivariate logistic regression analysis of postoperative POCD occurrence

The univariate logistic regression identified significant associations between POCD and CSI score, coronary heart disease, cerebrovascular disease, preoperative MMSE scores, intraoperative hemorrhage, and anesthesia time (all *p* < 0.05). To further evaluate the independent impact of central sensitization (CSI score) on the incidence of POCD, a multivariate logistic regression model was constructed. The analysis revealed that a CSI score of ≥40 (OR = 4.05, 95% CI = 1.52–11.54, *p* = 0.006), cerebrovascular disease (OR = 4.33, 95% CI = 1.09–18.68, *p* = 0.026), preoperative MMSE score < 27 (OR = 1.81, 95% CI = 1.04–3.26, *p* = 0.045), and intraoperative hemorrhage ≥200 mL (OR = 5.07, 95% CI = 1.58–20.14, *p* = 0.011) were significantly associated with a higher risk of POCD in TKA patients after adjusting for confounding factors (Table 4).

**Table 4:** Univariate and multivariate logistic regression of postoperative POCD occurrence.

Variables	Univariate logistic regression		Multivariate logistic regression	
	OR (95% CI)	*p* value	OR (95% CI)	*p* value
CSI ≥ 40	3.09 (1.33–7.43)	**0.010**	4.05 (1.52–11.54)	**0.006**
Age	1.03 (0.95–1.12)	0.461		
Gender	0.84 (0.36–1.92)	0.673		
BMI	0.99 (0.89–1.10)	0.848		
Educational level below high school	0.41 (0.15–1.00)	0.062		
ASA classification				
I	0.47 (0.07–1.80)	0.336		
II	1.23 (0.52–3.09)	0.643		
III	1.13 (0.41–2.85)	0.806		
Smoking	1.90 (0.45–2.44)	0.149		
Drinking	0.45 (0.16–1.14)	0.110		
Duration of KOA	1.04 (0.99–1.10)	0.130		
Combined underlying diseases				
Hypertension	1.29 (0.54–3.01)	0.553		
Hyperlipidemia	0.48 (0.11–1.51)	0.255		
Diabetes	1.63 (0.66–3.88)	0.278		
Coronary heart disease	4.17 (1.27–13.77)	**0.018**	2.49 (0.58–10.92)	0.216
Cerebrovascular disease	3.89 (1.27–11.64)	**0.015**	4.33 (1.09–18.68)	**0.026**
Chronic obstructive pulmonary	2.10 (0.81–5.21)	0.116		
Preoperative hemoglobin	1.02 (0.99–1.05)	0.139		
Anemia	0.81 (0.04–5.28)	0.848		
Preoperative MMSE scores <27	2.28 (1.40–3.93)	**0.002**	1.81 (1.04–3.26)	**0.045**
Operation time ≥ 120 min	1.60 (0.55–5.83)	0.423		
Intraoperative hemorrhage≥200 mL	10.08 (2.01–183.6)	**0.026**	10.51 (1.79–208.3)	**0.040**
Anesthesia type				
General anesthesia	1.74 (0.75–4.22)	0.206		
Neuraxial anesthesia	0.37 (0.12–0.98)	0.063		
Combination anesthesia	1.55 (0.46–4.53)	0.440		
Anesthesia time ≥ 150 min	6.00 (1.67–38.49)	**0.019**	1.77 (1.03–33.20)	0.077

Bold indicates statistical significance *p* < 0.05.

CSI, central sensitization inventory; BMI, body mass index; ASA, American society of anesthesiologists; KOA, knee osteoarthritis; MMSE, Mini-Mental State Examination.

## 4 Discussion

This study demonstrates that CS is a significant risk factor for POCD following TKA. A retrospective analysis of 142 patients revealed that 30.91% of the CS group experienced POCD, compared to 12.64% in the non-CS group. Additionally, the CS group exhibited lower MMSE scores at multiple postoperative time points, along with lower pain and quality of life scores in the KOOS subdomains compared to the non-sensitized group. Logistic regression analysis further indicated that even after accounting for potential confounders, a higher CSI score (≥40) remained an independent risk factor for POCD. This finding clearly underscores the critical role of CS in impairing neurocognitive recovery following TKA.

TKA is one of the most cost-effective surgeries in orthopedics. Despite its high success rate, postoperative complications, especially POCD, are increasingly concerning, with rates reported to range from 19.4 to 72.0% 1 week postoperatively (4). Although the precise mechanisms underlying POCD remain unclear, several risk factors are known to be closely associated, including lower educational level (29), older age (30), alcohol use disorder (31, 32), preexisting cognitive impairment (33, 34). This study is the first to reveal a close link between CS and POCD, suggesting that CS not only affects postoperative pain management but may also exacerbate cognitive decline following TKA.

CS refers to a persistent and exaggerated response of the central nervous system to painful stimuli, characterized by a lowered pain threshold and abnormal pain perception. Recent studies have demonstrated that CS not only alters pain perception but may also impair cognitive function through various mechanisms (10). Neuroinflammation is considered a key pathophysiological foundation of CS. In chronic pain conditions, pro-inflammatory signals released from peripheral tissues can penetrate the blood–brain barrier, activating microglia and astrocytes, which in turn heightens neuronal excitability, leading to neuronal damage and apoptosis, potentially affecting cognitive function (35, 36). TKA is a highly invasive procedure often accompanied by severe postoperative pain and inflammation. During this process, the release of pro-inflammatory cytokines, such as IL-6, TNF-*α*, and IL-1β, exacerbates the effects of CS, potentially contributing to the development of POCD (37, 38). This mechanism may explain the occurrence of POCD in TKA patients, and the impact is likely to be more pronounced in those with CS. The results of this study’s MMSE assessments further support this hypothesis, indicating that CS may negatively affect postoperative cognitive recovery through similar mechanisms. We observed significant declines in MMSE scores on postoperative days 1and 3 compared to preoperative scores in both the CS and control groups, suggesting marked cognitive deterioration in the acute phase of TKA, which aligns with previous research. Additionally, MMSE scores in the CS group were significantly lower than those in the control group on postoperative days 1, 3, and 7, indicating a notable delay in cognitive recovery during the acute postoperative period among CS patients. This trend persisted through postoperative day 30, suggesting that CS has a sustained negative impact on long-term cognitive recovery. However, the potential impact of the learning effect could not be ignored. Conducting the MMSE assessment five times within 1 month might have led to patients becoming familiar with the test, artificially improving scores without truly reflecting cognitive recovery. Although we implemented standardized testing procedures and multidimensional data analyses to minimize the learning effect, it is not entirely possible to rule out its influence on the results. Furthermore, as this is a retrospective study, we were unable to directly verify or quantify the existence of the learning effect through experimental design. Future research should consider longer intervals between assessments or incorporate more complex cognitive tests to reduce the impact of the learning effect.

The KOOS results further demonstrated the negative impact of CS on postoperative recovery following TKA. Repeated measures ANOVA revealed that the CS group had significantly lower pain and quality of life scores compared to the non-sensitized group, while no significant differences were observed in the subdomains of symptoms, daily living activities, or sports/recreation. This suggests that CS primarily affects pain perception and quality of life, rather than other functional domains of the knee. Moreover, time had a significant main effect across all KOOS subdomains, indicating that overall knee function improved postoperatively for all patients. However, the interaction between time and group was significant for pain and quality of life scores, indicating that the trajectories of postoperative pain relief and quality of life recovery differed significantly between the high-sensitization and low-sensitization groups. Notably, by postoperative days 7 and 30, the CS group showed a marked delay in pain perception relief and quality of life improvement. This suggests that CS not only exacerbates postoperative pain perception but may also hinder the recovery of knee function by affecting patients’ psychological states and fear of physical activity, thereby exerting a prolonged negative effect on long-term quality of life. Consequently, during postoperative recovery in CS patients, greater emphasis should be placed on pain management and quality of life enhancement.

Notably, in the univariate and multivariate logistic regression analyses, even after adjusting for multiple confounding factors, central sensitization (CSI score ≥ 40) was identified as a significant risk factor for POCD following TKA, further emphasizing the critical role of CS in the development of POCD. Additionally, patients with cerebrovascular disease, intraoperative hemorrhage exceeding 200 mL and preoperative MMSE scores below 27 were significantly associated with an increased risk of POCD, suggesting that underlying cerebrovascular conditions, physiological stressors (such as intraoperative blood loss) and baseline cognitive function are key contributors to the occurrence of POCD. Consistent with previous research (2, 39), we speculate that patients with central sensitization may experience a heightened stress response during surgery, potentially leading to increased blood loss and a compounded neurocognitive burden. This finding also underscores the necessity of a thorough preoperative assessment, including screening for cognitive impairment, and cerebrovascular health, as well as meticulous intraoperative management for patients at risk of central sensitization to mitigate these adverse outcomes.

Although this study provides new evidence for understanding the association between CS and POCD, several limitations should be acknowledged. First, as a retrospective study, this research can only reveal correlations between patients with more pronounced CS symptoms and POCD, without establishing causality. Second, although multiple potential confounding factors were adjusted for, other factors not accounted for in the study-such as postoperative infections, preoperative and intraoperative drug use, emotional disorders like depression and anxiety, and sleep disturbances-may also influence the occurrence of POCD. Third, conducting the MMSE assessment five times within 1 month may have introduced a learning effect, where patients’ familiarity with the test could artificially improve scores rather than reflect true cognitive recovery. Moreover, the MMSE may lack the sensitivity required to identify nuanced cognitive changes, potentially affecting the accuracy of POCD evaluation. Future studies should consider adopting more detailed neuropsychological assessment tools to comprehensively evaluate specific cognitive domains. Fourth, the sample size of this study was relatively small, and the follow-up points were limited to postoperative days 1, 3, 7, and 30. The short follow-up duration and small sample size may introduce bias, and the findings should therefore be interpreted with caution. Finally, the study did not utilize imaging techniques, such as structural MRI, FDG-PET, or resting-state fMRI, nor were biomarkers employed to explore the impact of CS on patients (40). This limits the understanding of the neural mechanisms underlying POCD and hinders the development of personalized treatment strategies.

## 5 Conclusion

This study suggests that CS may be a novel risk factor for POCD following TKA, and may adversely affect postoperative recovery in terms of cognitive function, pain, and quality of life. Preoperative assessment of CS, particularly through individualized management of patients with chronic pain, may help reduce the incidence of POCD and improve overall postoperative outcomes after TKA. Future prospective, multicenter studies are needed to validate these findings and explore additional interventions to mitigate the negative impact of CS on postoperative cognitive function.

## Data Availability

The original contributions presented in the study are included in the article/supplementary material, further inquiries can be directed to the corresponding author.
